# Crystal structure and Hirshfeld surface analysis of di­aqua­bis­(isonicotinamide-κ*N*)bis­(2,4,6-tri­methyl­benzoato-κ*O* ^1^)nickel(II) dihydrate

**DOI:** 10.1107/S205698901701060X

**Published:** 2017-07-21

**Authors:** Tuncer Hökelek, Gizem Sertkaya, Ezgi Ay, Safiye Özkaya, Hacali Necefoğlu

**Affiliations:** a Department of Physics, Hacettepe University, 06800 Beytepe, Ankara, Turkey; b Department of Chemistry, Kafkas University, 36100 Kars, Turkey; c Department of Chemistry, Kafkas University, 36100 Kars, Turkey, International Scientific Research Centre, Baku State University, 1148 Baku, Azerbaijan

**Keywords:** crystal structure, nickel(II), coordination compound, benzoic acid, nicotinamide

## Abstract

In the title Ni^II^ complex, [Ni(C_10_H_11_O_2_)_2_(C_6_H_6_N_2_O)_2_(H_2_O)_2_]·2H_2_O, the divalent Ni ion occupies a crystallographically imposed centre of symmetry and is coordinated by two O atoms from the carboxyl­ate groups of two 2,4,6-tri­methyl­benzoate (TMB) ligands [Ni—O = 2.0438 (12) Å], two N atoms from the pyridyl groups of two isonicotinamide (INA) ligands [Ni—N = 2.1506 (15) Å] and two water mol­ecules [Ni—O = 2.0438 (12) Å] in a slightly distorted octa­hedral geometry. The coordinating water mol­ecules are hydrogen bonded to the non-coordinating carboxyl­ate O atom of the TMB ligand [O⋯O = 2.593 (3) Å], enclosing an *S*(6) hydrogen-bonding motif. Two solvent water mol­ecules are also present in the formula unit. In the crystal, a network of inter­molecular N—H⋯O and O—H⋯O hydrogen bonds link the complexes into a three-dimensional array. Hirshfeld surface analysis indicates that the most important contributions for the crystal packing are from H⋯H (59.8%), O⋯H/H⋯O (20.2%) and C⋯H/H⋯C (13.7%) inter­actions.

## Abstract 2

In the title Ni^II^ complex, the divalent Ni ion occupies a crystallographically imposed centre of symmetry and is coordinated by two O atoms from the carboxyl­ate groups of two 2,4,6-tri­methyl­benzoate ligands, two N atoms from the pyridyl groups of two isonicotinamide ligands and two water mol­ecules in a slightly distorted octa­hedral geometry. Hirshfeld surface analysis indicates that the most important contributions for the crystal packing are from H⋯H (59.8%), O⋯H/H⋯O (20.2%) and C⋯H/H⋯C (13.7%) inter­actions.

## Chemical context   

Nicotinamide (NA) is a derivative of nicotinic acid, also called niacin. A deficiency in this vitamin leads to loss of copper from the body, giving rise to a condition known as pellagra disease. Victims of pellagra show unusually high serum and urinary copper levels (Krishnamachari, 1974 ▸). The crystal structure of NA was first determined in 1954 (Wright & King, 1954 ▸). The NA ring is the reactive part of nicotinamide adenine dinucleotide (NAD) and its phosphate (NADP), which are the major electron carriers in many biological oxidation–reduction reactions (You *et al.*, 1978 ▸). Another nicotinic acid derivative, *N*,*N*-di­ethyl­nicotinamide (DENA), is an important respiratory stimulant (Bigoli *et al.*, 1972 ▸). Transition-metal complexes with ligands of biochemical inter­est, such as imidazole and some N-protected amino acids, often show inter­esting physical and/or chemical properties, which lead to applications in biological systems (Antolini *et al.*, 1982 ▸). There have been many reports of the crystal structures of metal complexes with benzoic acid derivatives, which are of inter­est because of the number of different coordination modes exhibited by the carb­oxy­lic acid groups. These include Co and Cd complexes with 4-amino­benzoic acid (Chen & Chen, 2002 ▸; Amiraslanov *et al.*, 1979 ▸; Hauptmann *et al.*, 2000 ▸), Co complexes with benzoic acid (Catterick *et al.*, 1974 ▸), 4-nitro­benzoic acid (Nadzhafov *et al.*, 1981 ▸) and phthalic acid (Adiwidjaja *et al.*, 1978 ▸) and Cu complexes with 4-hydro­chloro­benzoic acid (Shnulin *et al.*, 1981 ▸). Mn complexes closely related to the title compound have also been reported, *e.g.* di­aqua­bis­(4-nitro­benzoato)bis­(1*H*-1,2,4-triazol-3-amine)­manganese(II) (Zhang *et al.*, 2013 ▸) and di­aqua­bis­(1*H*-imidazole)­bis­(4-nitro­benzoato)manganese(II) (Xu & Xu, 2004 ▸).
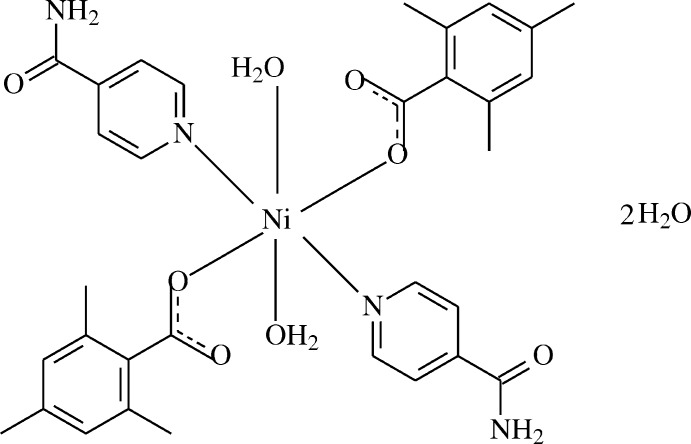


The crystal structures of anhydrous zinc(II) carboxyl­ates are diverse and include one-dimensional (Guseinov *et al.*, 1984 ▸; Clegg *et al.*, 1986*a* ▸), two-dimensional (Clegg *et al.*, 1986*b* ▸, 1987 ▸) and three-dimensional (Capilla & Aranda, 1979 ▸) polymeric motifs of different types, while discrete monomeric complexes with octa­hedral or tetra­hedral coordination geometry are found if water or other donor mol­ecules are coordinated to Zn (van Niekerk *et al.*, 1953 ▸; Usubaliev *et al.*, 1992 ▸). Pertinent to the present work, the structure–function–coordination relationships of the aryl­carboxyl­ate ion in Zn^II^ complexes of benzoic acid derivatives have been studied and shown to depend on the nature and position of the substituted groups on the benzene ring, the nature of the additional ligand, mol­ecule or solvent, and the pH and temperature of synthesis (Shnulin *et al.*, 1981 ▸; Nadzhafov *et al.*, 1981 ▸; Antsyshkina *et al.*, 1980 ▸; Adiwidjaja *et al.*, 1978 ▸; Catterick *et al.*, 1974 ▸).

The structures of a number of mononuclear complexes of divalent transition-metal ions with both nicotinamide (NA) and benzoic acid derivatives as ligands have been previously reported and include [Ni(C_7_H_4_ClO_2_)_2_(C_6_H_6_N_2_O)_2_(H_2_O)_2_] [(II); Hökelek *et al.*, 2009 ▸], [Ni(C_8_H_7_O_2_)_2_(C_6_H_6_N_2_O)_2_(H_2_O)_2_] [(III); Necefoğlu *et al.*, 2010 ▸], [Ni(C_8_H_7_O_3_)_2_(C_6_H_6_N_2_O)_2_(H_2_O)_2_]·2(H_2_O) [(IV); Hökelek *et al.*, 2010 ▸], [Ni(C_8_H_5_O_3_)_2_(C_6_H_6_N_2_O)_2_(H_2_O)_2_] [(V); Sertçelik *et al.*, 2012 ▸], [Mn(C_7_H_4_NO_4_)_2_(C_6_H_6_N_2_O)_2_(H_2_O)_2_] [(VI); Aşkın *et al.*, 2016 ▸] and [Zn(C_8_H_8_NO_2_)_2_(C_6_H_6_N_2_O)_2_] [(VII); Tercan *et al.*, 2009 ▸]. In this work, to enable comparison with the above Ni^II^ compounds and develop structure–function–coordination relationships, we describe the synthesis of di­aqua­bis(iso­nicotinamide-κ*N*)bis­(2,4,6-tri­methyl­benzoato-κ*O* ^1^)nickel(II) dihydrate, [Ni(C_10_H_11_O_2_)_2_(C_6_H_6_N_2_O)_2_(H_2_O)_2_]·2H_2_O, and report its mol­ecular and crystal structures, along with a Hirshfeld surface analysis.

## Structural commentary   

**Figure 1:**
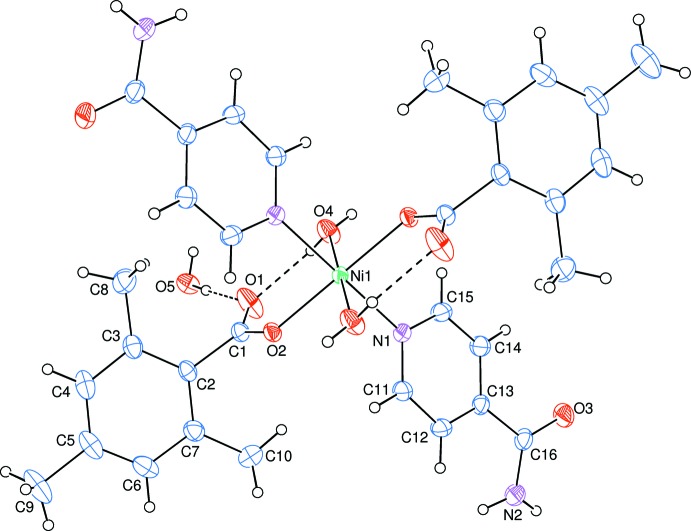
The mol­ecular structure of the title complex with the atom-numbering scheme. Unlabelled atoms are related to corresponding labelled ones by the symmetry operation (1 − *x*, −*y*, 1 − *z*). Displacement ellipsoids are drawn at the 50% probability level. O—H_coordW_⋯O_c_ and O—H_noncoordW_⋯O_c_ (c = carboxyl­ate, coordW = coordinating water and noncoordW = non-coordinating water) hydrogen bonds are shown as dashed lines.

The asymmetric unit of the mononuclear title compound (I) contains a Ni^II^ cation residing on a centre of symmetry, one 2,4,6-tri­methyl­benzoate (TMB) anion and one isonicotinamide (INA) anion, together with one coordinating and one non-coordinating water mol­ecule. The TMB and INA ligands coordinate in a monodentate manner (Fig. 1 ▸). In the complex, the Ni1 atom is in a slightly distorted octa­hedral environment and is coordinated by two carboxyl­ate O atoms (O2 and O2^i^) of the monodentate TMB anions, two coordinating water O atoms (O4 and O4^i^) and two pyridine N atoms (N1 and N1^i^) of the monodentate INA ligands at distances of 2.0438 (12), 2.0346 (14) and 2.1506 (15) Å, respectively [symmetry code: (i) 1 − *x*, −*y*, 1 − *z*] (Fig. 1 ▸). The non-coordinating oxygen atoms of the carboxyl­ate groups inter­act with the coordinating and non-coordinating water mol­ecules *via* short hydrogen bonds (Table 1 ▸, Fig. 1 ▸). Intra­molecular O—H_coordW_⋯O_c_ (coordW = coordinating water and c = carboxyl­ate) hydrogen bonds (Table 1 ▸) link H atoms of the coordinating water mol­ecules to the non-coordinating carboxyl­ate oxygen atoms, enclosing S(6) ring motifs (Fig. 1 ▸).

The near equalities of the C1—O1 [1.242 (2) Å] and C1—O2 [1.260 (2) Å] bonds in the carboxyl­ate groups indicate delocalized bonding arrangements, rather than localized single and double bonds. The O2—C1—O1 bond angle [124.52 (17)°] is comparable the corresponding values of 124.4 (2)° in (II), 124.67 (14)° in (III), 124.22 (11)° in (IV), 125.71 (10)° in (V), 126.0 (3)° in (VI) and 120.47 (15) and 123.17 (15)° in (VII), where the benzoate ions also coordinate the metal atoms monodentately. The Ni1 atom lies 0.3523 (1) Å below the planar (O1/O2/C1) carboxyl­ate group. In the TMB anion, the carboxyl­ate group is twisted away from the attached benzene, *A* (C2–C7), ring by 78.80 (14)°, while the benzene and pyridine, *B* (N1/C11–C15), rings are oriented at a dihedral angle of 24.33 (6)°.

## Supra­molecular features   

**Figure 2:**
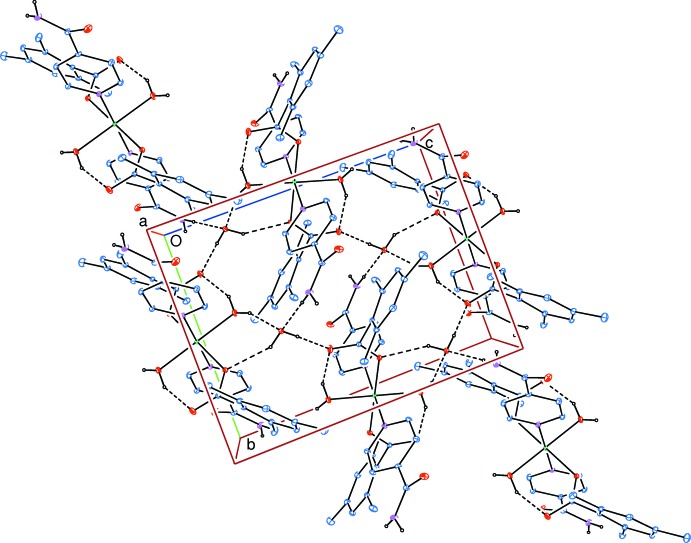
View of the hydrogen bonding and packing of the title complex along the *a* axis. Non-bonding H atoms have been omitted for clarity.

In the crystal structure, O—H_coordW_⋯O_noncoordW_, O—H_noncoordW_⋯O_c_, N—H_INA_⋯O_noncoordW_ and N—H_INA_⋯O_INA_ (INA = isonicotinamide and noncoordW = non-coordinating water) hydrogen bonds (Table 1 ▸) link the mol­ecules (Fig. 2 ▸) into networks parallel to [011], enclosing 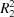
(6), 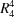
(19), 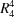
(26), 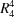
(28), 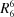
(32), 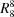
(28) and 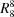
(32) ring motifs. The crystal structure is further stabilized by a weak C—H_INA_⋯O_noncoordW_ inter­action (Table 1 ▸).

## Hirshfeld surface analysis   

**Figure 3:**
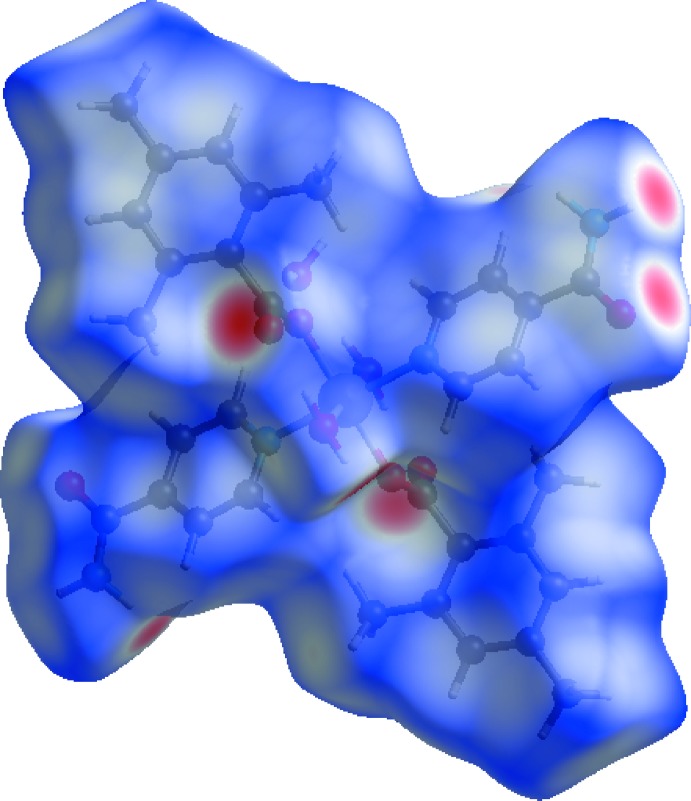
View of the three-dimensional Hirshfeld surface of the title complex plotted over *d*_norm_ in the range −0.7129 to 1.3644 au.

**Figure 4:**
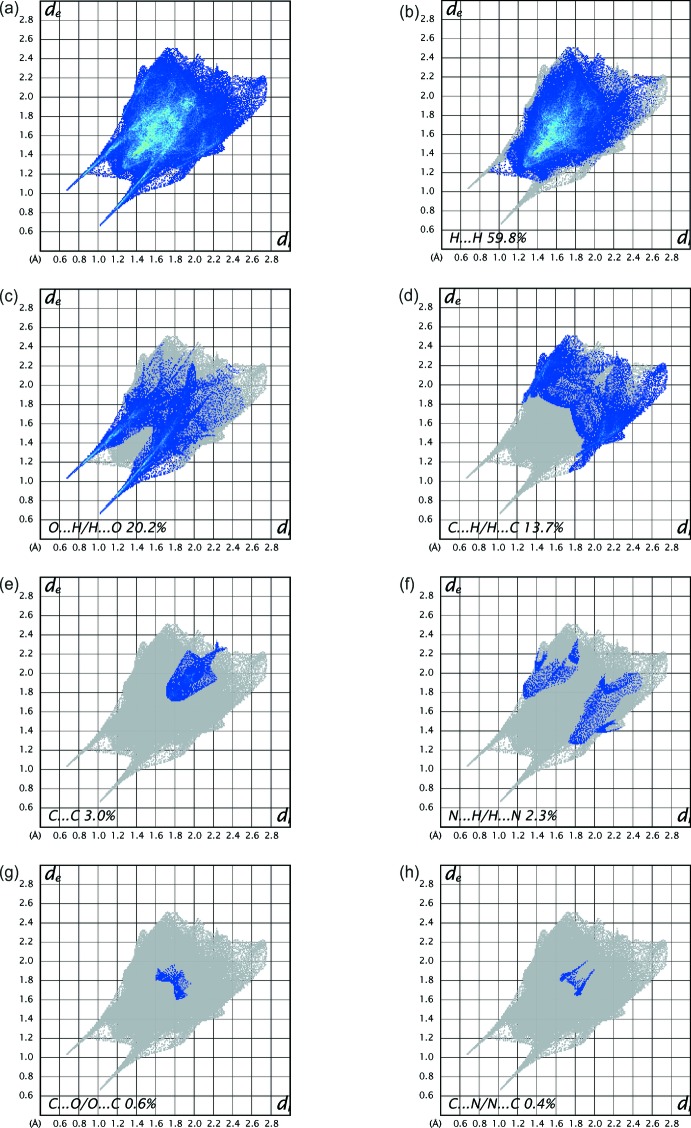
The full two-dimensional fingerprint plots from Hirshfeld analysis of the title complex, showing (*a*) all inter­actions, and delineated into (*b*) H⋯H, (*c*) O⋯H/H⋯O, (*d*) C⋯H/H⋯C, (*e*) C⋯C, (*f*) N⋯H/H⋯N, (*g*) C⋯O/O⋯C and (*h*) C⋯N/N⋯C inter­actions. The *d*_i_ and *d*_e_ values are the closest inter­nal and external distances (in Å) from given points on the Hirshfeld surface contacts.

**Figure 5:**
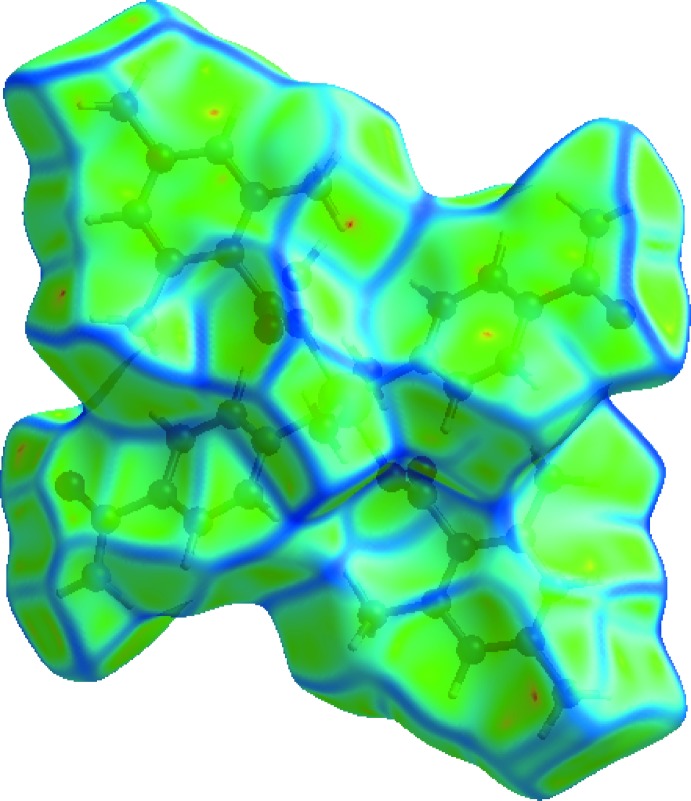
Hirshfeld surface of the title complex plotted over curvedness.

A Hirshfeld surface analysis (Hirshfeld, 1977 ▸; Spackman & Jayatilaka, 2009 ▸) of the title complex was carried out to investigate the locations of the atoms with potential to form hydrogen bonds and the qu­anti­tative ratios of these inter­actions. Conventional mapping of *d*_norm_ (Fig. 3 ▸), together with graphical representation of the Hirshfeld surface (Fig. 4 ▸) suggest the locations of the donors and acceptors of inter­molecular contacts, which are represented in Fig. 3 ▸ as bright-red spots near respective atoms. According to the analysis results, the most important inter­action is H⋯H contributing 59.8% to the overall crystal packing. The next most important inter­actions are O⋯H/H⋯O and C⋯H/H⋯C contributing 20.2% and 13.7%, respectively. The weakest inter­molecular contacts contributing to the cohesion of the structure are C⋯C, N⋯H/H⋯N, C⋯O/O⋯C and C⋯N/N⋯C, found to contribute only 3.0, 2.3, 0.6 and 0.4%, respectively. The overall two-dimensional fingerprint plot, Fig. 4 ▸ *a*, and those delineated into H⋯H, O⋯H/H⋯O, C⋯H/H⋯C, C⋯C, N⋯H/H⋯N, C⋯O/O⋯C and C⋯N/N⋯C contacts (McKinnon *et al.*, 2007 ▸) are illustrated in Fig. 4 ▸ *b*–*h*, respectively, together with their relative contributions to the Hirshfeld surface, where the significant O⋯H/H⋯O inter­actions are indicated by the pair of wings in the two-dimensional fingerprint plot with a prominent long spike at *d*_e_ + *d*_i_ ∼1.0 Å (Fig. 4 ▸ *c*). The presence of these inter­actions may also be shown by the Hirshfeld surface mapped as a function of curvedness (Fig. 5 ▸).

## Synthesis and crystallization   

The title compound was prepared by mixing solutions of NiSO_4_·6H_2_O (0.66 g, 2.5 mmol) in H_2_O (50 ml) and isonicotinamide (0.61 g, 5 mmol) in H_2_O (25 ml) with sodium 2,4,6-tri­methyl­benzoate (0.93 g, 5 mmol) in H_2_O (150 ml) at room temperature. The mixture was set aside to crystallize at ambient temperature for nine weeks and gave green single crystals (yield: 1.46 g, 83%). Combustion analysis: found; C, 54.70, H, 6.24; N, 8.13%. Calculated: C_32_H_42_N_4_NiO_10_ C, 54.80; H, 6.04; N, 7.99%. FT–IR: 3354, 3197, 2235, 1949, 1855, 1698, 1934, 1612, 1557, 1415, 1226, 1182, 1148, 1115, 1096, 1066, 1041, 1017, 985, 885, 855, 792, 772, 747, 682, 660, 638, 615, 520, 443 cm ^−1^.

## Refinement   

The experimental details including the crystal data, data collection and refinement are summarized in Table 2 ▸. H atoms of NH_2_ groups and water mol­ecules were located in difference Fourier maps and refined freely. The C-bound H atoms were positioned geometrically with C—H = 0.93 and 0.96 Å for aromatic and methyl H atoms, respectively, and constrained to ride on their parent atoms, with *U*_iso_(H) = *k* × *U*_eq_(C), where *k* = 1.5 for methyl H atoms and *k* = 1.2 for aromatic H atoms. The maximum and minimum residual density peaks were found at 0.83 and 0.78 Å from atoms O1 and O4, respectively.

## Supplementary Material

Crystal structure: contains datablock(s) I, global. DOI: 10.1107/S205698901701060X/cq2020sup1.cif

Structure factors: contains datablock(s) I. DOI: 10.1107/S205698901701060X/cq2020Isup2.hkl

CCDC reference: 1562879

Additional supporting information:  crystallographic information; 3D view; checkCIF report

## Figures and Tables

**Table 1:** Hydrogen-bond geometry (Å, °)

*D*—H⋯*A*	*D*—H	H⋯*A*	*D*⋯*A*	*D*—H⋯*A*
N2—H21⋯O5^i^	0.84 (3)	2.18 (3)	3.014 (3)	174 (2)
N2—H22⋯O3^ii^	0.83 (3)	2.21 (3)	3.043 (3)	177 (2)
O4—H41⋯O5^iii^	0.77 (3)	2.02 (3)	2.745 (2)	157 (3)
O4—H42⋯O1	0.81 (3)	1.85 (3)	2.593 (3)	151 (3)
O5—H51⋯O2^iv^	0.81 (3)	2.16 (3)	2.8804 (19)	148 (3)
O5—H52⋯O1	0.85 (3)	1.83 (3)	2.673 (2)	176 (2)
C12—H12⋯O5^i^	0.93	2.56	3.307 (2)	137

Symmetry codes: (i) 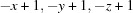; (ii) 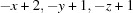; (iii) 
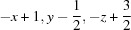
; (iv) 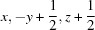.

**Table 2:** Experimental details

Crystal data	
Chemical formula	[Ni(C_10_H_11_O_2_)_2_(C_6_H_6_N_2_O)_2_(H_2_O)_2_]·2H_2_O
*M*_r_	701.41
Crystal system, space group	Monoclinic, *P*2_1_/*c*
Temperature (K)	296
*a*, *b*, *c* (Å)	14.0222 (3), 9.8275 (2), 13.0229 (3)
β (°)	105.645 (3)
*V* (Å^3^)	1728.11 (6)
*Z*	2
Radiation type	Mo *K*α
μ (mm^−1^)	0.62
Crystal size (mm)	0.45 × 0.30 × 0.28

Data collection	
Diffractometer	Bruker SMART BREEZE CCD
Absorption correction	Multi-scan (*SADABS*; Bruker, 2012 ▸)
*T*_min_, *T*_max_	0.767, 0.845
No. of measured, independent and observed [*I* > 2σ(*I*)] reflections	36737, 4290, 3618
*R*_int_	0.024
(sin θ/λ)_max_ (Å^−1^)	0.667

Refinement	
*R*[*F* ^2^ > 2σ(*F* ^2^)], *wR*(*F* ^2^), *S*	0.038, 0.103, 1.06
No. of reflections	4290
No. of parameters	241
H-atom treatment	H atoms treated by a mixture of independent and constrained refinement
Δρ_max_, Δρ_min_ (e Å^−3^)	0.57, −0.42

Computer programs: *APEX2* and *SAINT* (Bruker, 2012 ▸), *SHELXS97* and *SHELXL97* (Sheldrick, 2008 ▸), *ORTEP-3 for Windows* and *WinGX* (Farrugia, 2012 ▸) and *PLATON* (Spek, 2009 ▸).

## supplementary crystallographic information

### Crystal data

**Table:**

[Ni(C_10_H_11_O_2_)_2_(C_6_H_6_N_2_O)_2_(H_2_O)_2_]·2H_2_O	*F*(000) = 740
*M**_r_* = 701.41	*D*_x_ = 1.348 Mg m^−^^3^
Monoclinic, *P*2_1_/*c*	Mo *K*α radiation, λ = 0.71073 Å
*a* = 14.0222 (3) Å	Cell parameters from 9322 reflections
*b* = 9.8275 (2) Å	θ = 2.6–28.3°
*c* = 13.0229 (3) Å	µ = 0.62 mm^−^^1^
β = 105.645 (3)°	*T* = 296 K
*V* = 1728.11 (6) Å^3^	Block, translucent light blue
*Z* = 2	0.45 × 0.30 × 0.28 mm

### Data collection

**Table:**

Bruker SMART BREEZE CCD diffractometer	4290 independent reflections
Radiation source: fine-focus sealed tube	3618 reflections with *I* > 2σ(*I*)
Graphite monochromator	*R*_int_ = 0.024
φ and ω scans	θ_max_ = 28.3°, θ_min_ = 1.5°
Absorption correction: multi-scan (*SADABS*; Bruker, 2012)	*h* = −18→18
*T*_min_ = 0.767, *T*_max_ = 0.845	*k* = −13→12
36737 measured reflections	*l* = −17→17

### Refinement

**Table:**

Refinement on *F*^2^	Primary atom site location: structure-invariant direct methods
Least-squares matrix: full	Secondary atom site location: difference Fourier map
*R*[*F*^2^ > 2σ(*F*^2^)] = 0.038	Hydrogen site location: inferred from neighbouring sites
*wR*(*F*^2^) = 0.103	H atoms treated by a mixture of independent and constrained refinement
*S* = 1.06	*w* = 1/[σ^2^(*F*_o_^2^) + (0.0463*P*)^2^ + 1.1666*P*] where *P* = (*F*_o_^2^ + 2*F*_c_^2^)/3
4290 reflections	(Δ/σ)_max_ < 0.001
241 parameters	Δρ_max_ = 0.57 e Å^−^^3^
0 restraints	Δρ_min_ = −0.42 e Å^−^^3^

### Special details

**Table:**

Geometry. All esds (except the esd in the dihedral angle between two l.s. planes) are estimated using the full covariance matrix. The cell esds are taken into account individually in the estimation of esds in distances, angles and torsion angles; correlations between esds in cell parameters are only used when they are defined by crystal symmetry. An approximate (isotropic) treatment of cell esds is used for estimating esds involving l.s. planes.
Refinement. Refinement of F^2^ against ALL reflections. The weighted R-factor wR and goodness of fit S are based on F^2^, conventional R-factors R are based on F, with F set to zero for negative F^2^. The threshold expression of F^2^ > 2sigma(F^2^) is used only for calculating R-factors(gt) etc. and is not relevant to the choice of reflections for refinement. R-factors based on F^2^ are statistically about twice as large as those based on F, and R- factors based on ALL data will be even larger.

### Fractional atomic coordinates and isotropic or equivalent isotropic displacement parameters (Å^2^)

**Table:**

	*x*	*y*	*z*	*U*_iso_*/*U*_eq_	
Ni1	0.5000	0.0000	0.5000	0.02665 (10)	
O1	0.40085 (14)	0.2668 (2)	0.58794 (12)	0.0658 (5)	
O2	0.40320 (9)	0.15459 (13)	0.44114 (9)	0.0320 (3)	
O3	0.95561 (10)	0.34236 (17)	0.54825 (15)	0.0584 (4)	
O4	0.51379 (13)	0.0578 (2)	0.65335 (12)	0.0498 (4)	
H41	0.557 (2)	0.035 (3)	0.701 (2)	0.054 (8)*	
H42	0.490 (2)	0.133 (3)	0.653 (2)	0.073 (10)*	
O5	0.32828 (12)	0.41847 (15)	0.71977 (12)	0.0435 (3)	
H51	0.337 (2)	0.371 (3)	0.772 (2)	0.062 (8)*	
H52	0.349 (2)	0.371 (3)	0.676 (2)	0.066 (8)*	
N1	0.62368 (11)	0.12585 (16)	0.49343 (12)	0.0332 (3)	
N2	0.86640 (15)	0.4682 (2)	0.41092 (17)	0.0486 (4)	
H21	0.810 (2)	0.497 (2)	0.378 (2)	0.043 (7)*	
H22	0.915 (2)	0.520 (3)	0.420 (2)	0.054 (7)*	
C1	0.36860 (13)	0.24339 (18)	0.49098 (14)	0.0323 (4)	
C2	0.28216 (13)	0.32228 (18)	0.42324 (14)	0.0335 (4)	
C3	0.18589 (14)	0.2849 (2)	0.42355 (17)	0.0411 (4)	
C4	0.10724 (16)	0.3481 (2)	0.3512 (2)	0.0538 (6)	
H4	0.0429	0.3240	0.3504	0.065*	
C5	0.12132 (18)	0.4452 (3)	0.2806 (2)	0.0576 (6)	
C6	0.2168 (2)	0.4839 (2)	0.28483 (19)	0.0534 (6)	
H6	0.2268	0.5519	0.2392	0.064*	
C7	0.29841 (15)	0.4242 (2)	0.35545 (16)	0.0422 (4)	
C8	0.16723 (18)	0.1801 (3)	0.5003 (2)	0.0594 (6)	
H8A	0.2090	0.1984	0.5705	0.089*	
H8B	0.1817	0.0912	0.4780	0.089*	
H8C	0.0991	0.1841	0.5013	0.089*	
C9	0.0339 (3)	0.5094 (4)	0.2002 (3)	0.0906 (12)	
H9A	0.0563	0.5862	0.1675	0.136*	
H9B	−0.0142	0.5388	0.2358	0.136*	
H9C	0.0044	0.4438	0.1464	0.136*	
C10	0.4011 (2)	0.4727 (3)	0.3593 (2)	0.0700 (8)	
H10A	0.4484	0.4047	0.3928	0.105*	
H10B	0.4146	0.5557	0.3996	0.105*	
H10C	0.4057	0.4887	0.2881	0.105*	
C11	0.62245 (14)	0.2010 (2)	0.40774 (16)	0.0406 (4)	
H11	0.5659	0.1991	0.3505	0.049*	
C12	0.70084 (14)	0.2816 (2)	0.39966 (17)	0.0430 (4)	
H12	0.6966	0.3317	0.3380	0.052*	
C13	0.78570 (13)	0.28715 (19)	0.48380 (16)	0.0358 (4)	
C14	0.78705 (14)	0.2095 (2)	0.57270 (17)	0.0445 (5)	
H14	0.8426	0.2098	0.6310	0.053*	
C15	0.70590 (14)	0.1317 (2)	0.57467 (16)	0.0431 (4)	
H15	0.7083	0.0807	0.6354	0.052*	
C16	0.87691 (14)	0.3701 (2)	0.48379 (18)	0.0418 (4)	

### Atomic displacement parameters (Å^2^)

**Table:**

	*U*^11^	*U*^22^	*U*^33^	*U*^12^	*U*^13^	*U*^23^
Ni1	0.02458 (15)	0.03057 (17)	0.02553 (15)	0.00352 (11)	0.00801 (11)	0.00199 (11)
O1	0.0765 (11)	0.0751 (12)	0.0351 (7)	0.0410 (10)	−0.0032 (7)	−0.0140 (8)
O2	0.0322 (6)	0.0340 (6)	0.0301 (6)	0.0082 (5)	0.0092 (5)	0.0018 (5)
O3	0.0294 (7)	0.0551 (10)	0.0858 (12)	−0.0053 (6)	0.0070 (7)	0.0178 (9)
O4	0.0576 (10)	0.0612 (11)	0.0275 (7)	0.0245 (8)	0.0059 (6)	−0.0006 (7)
O5	0.0552 (9)	0.0393 (8)	0.0330 (7)	0.0042 (6)	0.0067 (6)	−0.0031 (6)
N1	0.0285 (7)	0.0329 (8)	0.0388 (8)	−0.0003 (6)	0.0105 (6)	0.0041 (6)
N2	0.0338 (9)	0.0453 (10)	0.0666 (12)	−0.0084 (8)	0.0135 (8)	0.0108 (9)
C1	0.0318 (8)	0.0314 (8)	0.0334 (8)	0.0046 (7)	0.0085 (7)	−0.0008 (7)
C2	0.0341 (9)	0.0327 (9)	0.0328 (8)	0.0089 (7)	0.0073 (7)	−0.0035 (7)
C3	0.0358 (9)	0.0368 (10)	0.0482 (11)	0.0035 (8)	0.0067 (8)	−0.0054 (8)
C4	0.0344 (10)	0.0508 (13)	0.0680 (14)	0.0070 (9)	−0.0001 (9)	−0.0077 (11)
C5	0.0528 (13)	0.0517 (13)	0.0558 (13)	0.0194 (11)	−0.0068 (10)	−0.0016 (11)
C6	0.0654 (15)	0.0480 (13)	0.0434 (11)	0.0162 (10)	0.0087 (10)	0.0085 (9)
C7	0.0453 (11)	0.0434 (11)	0.0391 (10)	0.0097 (9)	0.0134 (8)	0.0032 (8)
C8	0.0480 (13)	0.0516 (14)	0.0794 (17)	−0.0059 (10)	0.0186 (12)	0.0066 (12)
C9	0.0697 (19)	0.093 (3)	0.085 (2)	0.0307 (17)	−0.0198 (17)	0.0133 (18)
C10	0.0578 (15)	0.0815 (19)	0.0781 (19)	0.0036 (13)	0.0313 (14)	0.0291 (15)
C11	0.0306 (9)	0.0450 (11)	0.0426 (10)	−0.0028 (8)	0.0038 (7)	0.0100 (8)
C12	0.0373 (10)	0.0421 (11)	0.0480 (11)	−0.0047 (8)	0.0090 (8)	0.0142 (9)
C13	0.0288 (8)	0.0304 (9)	0.0497 (10)	−0.0006 (7)	0.0132 (7)	0.0020 (8)
C14	0.0323 (9)	0.0494 (12)	0.0471 (11)	−0.0063 (8)	0.0025 (8)	0.0087 (9)
C15	0.0362 (9)	0.0495 (11)	0.0413 (10)	−0.0058 (8)	0.0063 (8)	0.0110 (9)
C16	0.0308 (9)	0.0366 (10)	0.0602 (12)	−0.0033 (7)	0.0161 (8)	0.0015 (9)

### Geometric parameters (Å, º)

**Table:**

Ni1—O2	2.0438 (12)	C4—H4	0.9300
Ni1—O2^i^	2.0438 (12)	C5—C6	1.379 (4)
Ni1—O4	2.0346 (14)	C5—C9	1.518 (3)
Ni1—O4^i^	2.0346 (14)	C6—H6	0.9300
Ni1—N1	2.1506 (15)	C7—C6	1.390 (3)
Ni1—N1^i^	2.1506 (15)	C7—C10	1.504 (3)
O1—C1	1.242 (2)	C8—H8A	0.9600
O2—C1	1.260 (2)	C8—H8B	0.9600
O3—C16	1.224 (2)	C8—H8C	0.9600
O4—H41	0.78 (3)	C9—H9A	0.9600
O4—H42	0.81 (3)	C9—H9B	0.9600
O5—H51	0.81 (3)	C9—H9C	0.9600
O5—H52	0.84 (3)	C10—H10A	0.9600
N1—C11	1.334 (2)	C10—H10B	0.9600
N1—C15	1.339 (2)	C10—H10C	0.9600
N2—C16	1.333 (3)	C11—C12	1.381 (3)
N2—H21	0.84 (3)	C11—H11	0.9300
N2—H22	0.84 (3)	C12—H12	0.9300
C1—C2	1.507 (2)	C13—C12	1.384 (3)
C2—C3	1.400 (3)	C13—C14	1.382 (3)
C2—C7	1.394 (3)	C13—C16	1.517 (2)
C3—C4	1.389 (3)	C14—H14	0.9300
C3—C8	1.506 (3)	C15—C14	1.377 (3)
C4—C5	1.375 (4)	C15—H15	0.9300
			
O2^i^—Ni1—O2	180.0	C5—C6—H6	119.1
O2—Ni1—N1	91.07 (5)	C7—C6—H6	119.1
O2^i^—Ni1—N1	88.93 (5)	C2—C7—C10	121.65 (19)
O2—Ni1—N1^i^	88.93 (5)	C6—C7—C2	118.4 (2)
O2^i^—Ni1—N1^i^	91.07 (5)	C6—C7—C10	119.9 (2)
O4—Ni1—O2	92.21 (6)	C3—C8—H8A	109.5
O4^i^—Ni1—O2	87.79 (6)	C3—C8—H8B	109.5
O4—Ni1—O2^i^	87.79 (6)	C3—C8—H8C	109.5
O4^i^—Ni1—O2^i^	92.21 (6)	H8A—C8—H8B	109.5
O4—Ni1—O4^i^	180.0	H8A—C8—H8C	109.5
O4—Ni1—N1	90.82 (7)	H8B—C8—H8C	109.5
O4^i^—Ni1—N1	89.18 (7)	C5—C9—H9A	109.5
O4—Ni1—N1^i^	89.18 (7)	C5—C9—H9B	109.5
O4^i^—Ni1—N1^i^	90.82 (7)	C5—C9—H9C	109.5
N1—Ni1—N1^i^	180.0	H9A—C9—H9B	109.5
C1—O2—Ni1	129.09 (11)	H9A—C9—H9C	109.5
Ni1—O4—H41	123 (2)	H9B—C9—H9C	109.5
Ni1—O4—H42	109 (2)	C7—C10—H10A	109.5
H41—O4—H42	120 (3)	C7—C10—H10B	109.5
H52—O5—H51	104 (3)	C7—C10—H10C	109.5
C11—N1—Ni1	121.52 (12)	H10A—C10—H10B	109.5
C11—N1—C15	116.82 (16)	H10A—C10—H10C	109.5
C15—N1—Ni1	121.66 (12)	H10B—C10—H10C	109.5
C16—N2—H21	121.3 (17)	N1—C11—C12	123.34 (18)
C16—N2—H22	114.1 (19)	N1—C11—H11	118.3
H21—N2—H22	119 (2)	C12—C11—H11	118.3
O1—C1—O2	124.52 (17)	C11—C12—C13	119.62 (18)
O1—C1—C2	120.95 (16)	C11—C12—H12	120.2
O2—C1—C2	114.53 (15)	C13—C12—H12	120.2
C3—C2—C1	119.14 (17)	C12—C13—C16	124.47 (18)
C7—C2—C1	119.82 (17)	C14—C13—C12	117.11 (17)
C7—C2—C3	120.86 (17)	C14—C13—C16	118.41 (17)
C2—C3—C8	121.44 (18)	C13—C14—H14	120.1
C4—C3—C2	118.0 (2)	C15—C14—C13	119.83 (18)
C4—C3—C8	120.5 (2)	C15—C14—H14	120.1
C3—C4—H4	118.9	N1—C15—C14	123.28 (18)
C5—C4—C3	122.2 (2)	N1—C15—H15	118.4
C5—C4—H4	118.9	C14—C15—H15	118.4
C4—C5—C6	118.6 (2)	O3—C16—N2	123.77 (19)
C4—C5—C9	121.0 (3)	O3—C16—C13	118.98 (18)
C6—C5—C9	120.5 (3)	N2—C16—C13	117.23 (18)
C5—C6—C7	121.8 (2)		
			
O4—Ni1—O2—C1	−1.87 (16)	C7—C2—C3—C4	2.7 (3)
O4^i^—Ni1—O2—C1	178.13 (16)	C7—C2—C3—C8	−176.8 (2)
N1—Ni1—O2—C1	−92.74 (15)	C1—C2—C7—C6	172.41 (18)
N1^i^—Ni1—O2—C1	87.26 (15)	C1—C2—C7—C10	−9.2 (3)
O2—Ni1—N1—C11	−45.97 (15)	C3—C2—C7—C6	−2.7 (3)
O2^i^—Ni1—N1—C11	134.03 (15)	C3—C2—C7—C10	175.6 (2)
O2—Ni1—N1—C15	134.61 (16)	C2—C3—C4—C5	−0.2 (3)
O2^i^—Ni1—N1—C15	−45.39 (16)	C8—C3—C4—C5	179.3 (2)
O4—Ni1—N1—C11	−138.19 (16)	C3—C4—C5—C6	−2.3 (4)
O4^i^—Ni1—N1—C11	41.81 (16)	C3—C4—C5—C9	178.1 (3)
O4—Ni1—N1—C15	42.39 (16)	C4—C5—C6—C7	2.3 (4)
O4^i^—Ni1—N1—C15	−137.61 (16)	C9—C5—C6—C7	−178.1 (3)
Ni1—O2—C1—O1	12.8 (3)	C2—C7—C6—C5	0.2 (3)
Ni1—O2—C1—C2	−167.00 (12)	C10—C7—C6—C5	−178.2 (2)
Ni1—N1—C11—C12	−178.99 (16)	N1—C11—C12—C13	−0.4 (3)
C15—N1—C11—C12	0.5 (3)	C14—C13—C12—C11	0.3 (3)
Ni1—N1—C15—C14	179.01 (17)	C16—C13—C12—C11	179.13 (19)
C11—N1—C15—C14	−0.4 (3)	C12—C13—C14—C15	−0.3 (3)
O1—C1—C2—C3	−81.0 (3)	C16—C13—C14—C15	−179.2 (2)
O1—C1—C2—C7	103.8 (2)	C12—C13—C16—O3	−161.4 (2)
O2—C1—C2—C3	98.9 (2)	C12—C13—C16—N2	17.2 (3)
O2—C1—C2—C7	−76.3 (2)	C14—C13—C16—O3	17.4 (3)
C1—C2—C3—C4	−172.43 (18)	C14—C13—C16—N2	−164.0 (2)
C1—C2—C3—C8	8.1 (3)	N1—C15—C14—C13	0.4 (4)

Symmetry code: (i) −*x*+1, −*y*, −*z*+1.

### Hydrogen-bond geometry (Å, º)

**Table:**

*D*—H···*A*	*D*—H	H···*A*	*D*···*A*	*D*—H···*A*
N2—H21···O5^ii^	0.84 (3)	2.18 (3)	3.014 (3)	174 (2)
N2—H22···O3^iii^	0.83 (3)	2.21 (3)	3.043 (3)	177 (2)
O4—H41···O5^iv^	0.77 (3)	2.02 (3)	2.745 (2)	157 (3)
O4—H42···O1	0.81 (3)	1.85 (3)	2.593 (3)	151 (3)
O5—H51···O2^v^	0.81 (3)	2.16 (3)	2.8804 (19)	148 (3)
O5—H52···O1	0.85 (3)	1.83 (3)	2.673 (2)	176 (2)
C12—H12···O5^ii^	0.93	2.56	3.307 (2)	137

Symmetry codes: (ii) −*x*+1, −*y*+1, −*z*+1; (iii) −*x*+2, −*y*+1, −*z*+1; (iv) −*x*+1, *y*−1/2, −*z*+3/2; (v) *x*, −*y*+1/2, *z*+1/2.
